# Palmitoylated prolactin-releasing peptide treatment had neuroprotective but not anti-obesity effect in fa/fa rats with leptin signaling disturbances

**DOI:** 10.1038/s41387-022-00205-3

**Published:** 2022-05-19

**Authors:** Lucia Mráziková, Silvie Hojná, Andrea Pačesová, Lucie Hrubá, Veronika Strnadová, Barbora Neprašová, Blanka Železná, Jaroslav Kuneš, Lenka Maletínská

**Affiliations:** 1 Institute of Organic Chemistry and Biochemistry CAS, Prague, Czech Republic; 2 First Faculty of Medicine, Charles University, Prague, Czech Republic; 3 Institute of Physiology CAS, Prague, Czech Republic

**Keywords:** Diseases of the nervous system, Drug discovery

## Abstract

**Background/Objective:**

Anorexigenic palmitoylated prolactin-releasing peptide (palm^11^-PrRP) is able to act centrally after peripheral administration in rat and mouse models of obesity, type 2 diabetes mellitus and/or neurodegeneration. Functional leptin and intact leptin signaling pathways are necessary for the body weight reducing and glucose tolerance improving effect of palm^11^-PrRP. We have previously shown that palm^11^-PrRP31 had glucose-lowering properties but not anti-obesity effect in Koletsky rats with leptin signaling disturbances, so improvements in glucose metabolism appear to be completely independent of leptin signaling. The purpose of this study was to describe relationship between metabolic and neurodegenerative pathologies and explore if palm^11^-PrRP31 could ameliorate them in obese fa/fa rat model with leptin signaling disruption.

**Subject/Methods:**

The fa/fa rats and their age-matched lean controls at the age 32 weeks were used for this study. The rats were infused for 2 months with saline or palm^11^-PrRP31 (*n* = 7–8 per group) at a dose of 5 mg/kg per day using Alzet osmotic pumps. During the dosing period food intake and body weight were monitored. At the end of experiment the oral glucose tolerance test was performed; plasma and tissue samples were collected and arterial blood pressure was measured. Then, markers of leptin and insulin signaling, Tau phosphorylation, neuroinflammation, and synaptogenesis were measured by western blotting and immunohistochemistry.

**Results:**

Fa/fa rats developed obesity, mild glucose intolerance, and peripheral insulin resistance but not hypertension while palm^11^-PrRP31 treatment neither lowered body weight nor attenuated glucose tolerance but ameliorated leptin and insulin signaling and synaptogenesis in hippocampus.

**Conclusion:**

We demonstrated that palm^11^-PrRP31 had neuroprotective features without anti-obesity and glucose lowering effects in fa/fa rats. This data suggest that this analog has the potential to exert neuroprotective effect despite of leptin signaling disturbances in this rat model.

## Introduction

The incidence of so-called diseases of civilization, such as obesity, insulin resistance (IR), type 2 diabetes mellitus (T2DM), and hypertension, is constantly rising, creating major health problems for developed and also developing countries [1–3]. Unfortunately, an unsatisfactory number of anti-obesity drugs are currently available. This dearth is related to the fact that a broad range of factors have been implicated in the progression of obesity and related diseases; therefore, it is difficult to find a suitable animal model with similar pathology to that of humans. It was suggested that obesity, together with T2DM and IR, can play a significant role in the pathology of neurodegenerative disorders, including Alzheimer’s disease (AD) [4]. The accuracy of this description is supported by the fact that cerebral glucose utilization and energy metabolism worsen with the progression of cognitive impairment, and the accumulation of amyloid β (Aβ) and Tau protein hyperphosphorylation, two main hallmarks of AD, are increased. Since increased Tau hyperphosphorylation was found in db/db mice with a nonfunctional leptin receptor [5], leptin was determined to play an important role in Tau pathology [1, 6].

Zucker fatty (fa/fa) rats are a model of genetic obesity. The single nucleotide mutation of the *fa* gene (Gln269Pro) leads to a mutation in the extracellular domain of all isoforms of the leptin Ob-R receptor that is critical for the obese phenotype [7–9]. As a consequence, fa/fa animals have elevated plasma leptin levels and are resistant to exogenous leptin administration [10]. The obesity of fa/fa rats was correlated with hyperphagia and decreased energy expenditure [11]. Moreover, fa/fa animals with normoglycemia and high lipid levels were found to exhibit IR, as shown by increased plasma cholesterol (CHOL), triglycerides (TG), and free fatty acid (FFA) levels compared to lean controls [12]. Multiple abnormalities in the cellular physiology of insulin-mediated glucose clearance have been associated with IR, including alterations in insulin binding and cellular signaling at its receptor, glucose metabolism, and glucose transport [13, 14].

Although the mechanism by which diabetes reduces cognitive function is not completely clear, research in several rodent models has led to the identification of numerous correlations between hippocampal functional impairment, obesity, and diabetes [1, 15]. In our previous study with 12- and 33-week-old Zucker fa/fa rats, we showed that aging and obesity significantly contributed to increased peripheral IR, which further worsened the activation of the hippocampal insulin signaling cascade. This effect resulted in decreased phosphorylation at the inhibitory epitope Ser 9 of glycogen-synthase kinase 3β (GSK-3β), one of the main kinases of Tau protein phosphorylation. Subsequently, an increase in the pathological hyperphosphorylation of Tau protein was observed in the hippocampi of fa/fa rats; thus, peripheral IR resulted in central insulin resistance and Tau hyperphosphorylation [16]. Moreover, a balance between kinases and phosphatases regulates the Tau phosphorylation state. The main phosphatase is protein phosphatase 2A (PP2A), while cyclin-dependent kinase 5 (Cdk5) is another important Tau kinase previously shown to be involved in abnormal Tau phosphorylation and activated in the brains of AD patients [17–19]. Beside Aβ plaques and increased Tau phosphorylation, increased neuroinflammation manifested by increased level of astrocytes or microglia, and decreased synaptogenesis and neurogenesis were described to contribute to development of AD and other neurodegenerative disordes [20–22]. Subsequently, neuroinflammation was recently demonstrated in the Zucker fa/fa rats brain [23].

It has been recently suggested that some food intake(FI)-regulating peptides may be promising candidates for obesity and T2DM treatment and for alleviating cognitive deficits in neurodegenerative disorders [1]. One of these peptides is the anorexigenic prolactin-releasing peptide (PrRP), centrally released and acting to regulate energy metabolism in cooperation with leptin [24]. The lipidization of its N-terminus enables its central effect after peripheral administration in rodents [25, 26]. Recently we have developed a novel PrRP analog palmitoylated at position 11 (palm^11^-PrRP31) with improved bioavailability, demonstrated that it is able to bind to PrRP receptor with high affinity in vitro and shown that PrRP analog decreased body weight (BW) and food intake in DIO mice [27–29]. In our previous studies, we demonstrated that functional leptin and intact leptin signaling pathways are necessary for the BW-reducing properties of palmitoylated PrRP in ob/ob mice, which are unable to produce leptin [30]; in Koletsky obese rats, which lack functional leptin receptors [31]; and in Zucker diabetic fatty (ZDF) rats [32]. Moreover, palmitoylated PrRP (palm-PrRP31) analog has been shown to have both anti-obesity and glucose- lowering properties, and it has also been shown to act as a neuroprotective compound in mouse models of Aβ or Tau pathology [33, 34].

Therefore, we hypothesize that aged fa/fa rats might be a suitable model to study the relationship between metabolic and neurodegenerative disorders. The aim of this study was to examine whether obesity and diabetic metabolic parameters of fa/fa rats relate not only to IR and Tau hyperphosphorylation in the brain but also to impaired synaptogenesis and neuroinflammation which are important players in AD progression. Then, the impact of treatment with a palmitoylated PrRP analog (palm^11^-PrRP31) on all these parameters was studied to examine its potential neuroprotective effects in this model of leptin and insulin resistance.

## Material and methods

### Synthesis of PrRP analog

A human palmitoylated analog of PrRP (palm^11^-PrRP31) with the sequence SRTHRHSMEIK (N-γ-E(N-palmitoyl)) TPDINPAWYASRGIRPVGRF-NH_2_ was synthesized and purified at the Institute of Organic Chemistry and Biochemistry, Czech Academy of Sciences, Prague (CAS), Czech Republic, as previously described [35].

### Animals and diet

All animal experiments were performed following the ethical guidelines for work with animals by the Act of the Czech Republic No. 246/1992 and were approved by the Committee for Experiments with Laboratory Animals of the CAS.

Experiments were conducted on homozygous Zucker *fa/fa* male rats (fa/fa) and their lean counterparts *fa*/^+−^ (control) rats. Six-week-old male rats of both genotypes were purchased from Charles River (Lecco, Italy). The rats were provided with a standard Ssniff diet (Spezialdiäten GmbH, Soest, Germany) (58% carbohydrates, 9% fat, and 33% protein) and water ad libitum. Animals were on a 12:12-h light:dark cycle (lights on from 5:00) and maintained at a constant temperature of 22 ± 2 °C.

### Study design, drug administration, and dosing

**Fig. 1:**
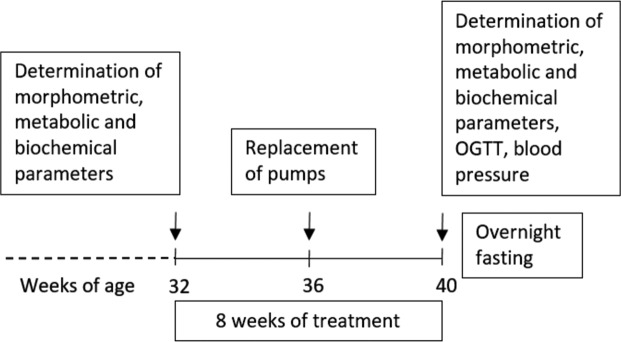
Experimental design.

The schema of the experimental design is shown in Fig. 1. Before the start of treatment, the BW and food intake (FI) of the fa/fa and control rats were monitored once per week. Fasted blood samples were collected from tail vessels of rats 32 weeks of age to determine the basic biochemical profile of the rat plasma. Beginning at 32 weeks of age, the fa/fa rats were infused for 2 months with palm^11^-PrRP31 dissolved in saline (fa/fa palm^11^-PrRP31 group, *n* = 8) at a dose of 5 mg/kg BW per day using Alzet osmotic pumps (Alzet, Cupertino, CA, USA), which were certified to infuse 6 µl of solution daily. The dose used in this study was chosen according to our previous studies [31, 32]. Two other groups, lean controls *fa*/^+−^ (control saline group, *n* = 8) and fa/fa rats (fa/fa saline group, *n* = 7), were infused with saline. All Alzet osmotic pumps were replaced after 4 weeks with new pumps. Alzet osmotic pumps were implanted intraperitoneally (IP) under short-term ether anesthesia. During the treatment, the BW and FI were measured twice per week. At the end of the experiment, the rats were fasted overnight, blood plasma was collected from tail vessels for determination of the biochemical parameters, and an oral glucose tolerance test (OGTT) was performed. Thereafter, arterial blood pressure was measured by direct puncture of the carotid artery under light ether anesthesia. The animals were sacrificed by decapitation, and tissue samples were collected. The brain, liver, kidney, and heart were dissected and kept frozen at −80 °C until use.

### Oral glucose tolerance test

Rats fasted overnight were subjected to OGTT. At time point 0, blood glucose was measured in the tail vessels blood, and then glucose, at a dose of 2 g/kg of BW, was administered perorally by gavage. Blood glucose concentrations in whole blood were determined using a glucometer (Arkray, Tokyo, Japan) at 15, 30, 60, 90, 120, and 180 min after glucose gavage. EDTA plasma samples were collected at 0, 30, 60, and 120 min intervals to determine insulin levels.

### Determination of biochemical parameters

Plasma insulin concentration was measured using a radioimmunoassay (RIA) kit (Millipore, St. Charles, MI, USA). Colorimetric assays were used to determine plasma levels of CHOL, TG (Erba Lachema, Brno, Czech Republic) and FFA (Roche, Mannheim, Germany). All measurements were performed according to the manufacturer’s instructions. Quantitative insulin sensitivity check index (QUICKI) was measured from fasting glucose and fasting insulin (QUICKI = 1/[(log *I*_0_) + (log *G*_0_)], where *I*_0_ is fasting insulin in µU/ml and *G*_0_ is fasting glucose in mg/dl).

### Drug exposure

The blood plasma concentration of palm^11^-PrRP31 at the end of the experiment was determined with a rat PrRP[1–31] EIA high-sensitivity kit (Peninsula Laboratories, San Carlos, CA, USA) according to the manufacturer’s instructions.

### Western blot analysis

Hippocampal samples were processed, and western blotting (WB) was performed as previously described [30]. The primary antibodies used are shown in Supplementary Table 1. The following secondary antibodies were used: anti-mouse or anti-rabbit IgG HRP-linked antibody (both Cell Signaling Technology, Beverly, MA, USA).

### Chromogenic immunohistochemistry (IHC)

One-half of the rat brains were processed as previously described [33]. The sections were incubated in anti-ionized calcium-binding adaptor molecule 1 (Iba1) (Wako, Osaka, Japan), anti-glial fibrillary acidic protein (GFAP) (Thermo Fisher Scientific, Waltham, MA, USA) or anti-doublecortin (DCX) (Cell Signaling Technology) primary rabbit antibodies. A biotinylated goat anti-rabbit secondary antibody was used for chromogenic IHC.

### Statistical analyses

The data are presented as the means ± SEM as analyzed with GraphPad Software (San Diego, CA, USA). The data were evaluated by *t*-test and one-way or two-way ANOVA followed by Bonferroni post hoc test. *P* < 0.05 was considered statistically significant.

## Results

### Palm^11^-PrRP31 had no effect on morphometric and metabolic parameters

The BW was significantly higher in the fa/fa rats compared to controls before treatment, at 32 weeks of age. At the end of the experiment (40 weeks of age), the BW and liver weight were also increased in fa/fa rats (Table 1). Treatment with palm^11^-PrRP31 did not significantly decrease the BW of the fa/fa rats. At age 32 and 40 weeks, the fa/fa rats had a significantly higher fasted glucose than the age-matched controls. After treatment with palm^11^-PrRP31, fa/fa rats had slightly increased glucose level, but still normoglycemic [31]. Plasma levels of insulin, leptin, CHOL, and TG were significantly higher in the fa/fa group than in the control group before the treatment and at the end of the experiment. Moreover, QUICKI was significantly decreased in fa/fa saline rats compared to lean controls. Treatment with palm^11^-PrRP31 had no effect on the measured metabolic parameters (Table 1). There were no significant changes in blood pressure among the groups (Table 1). The plasma concentration of palm^11^-PrRP31 in the fa/fa palm^11^-PrRP31 rats was 30.9 ± 8.7 ng/ml (*n* = 8) at the end of the experiment.

**Table 1:** Metabolic parameters in fasted blood plasma and in liver, organs weight and blood pressure of control and fa/fa group before treatment (32 weeks of age) and at the end of experiment (40 weeks of age) in control saline and fa/fa treated with saline or palm^11^-PrRP31.

Age	32 weeks		40 weeks		
Group	Control	fa/fa	Control saline	fa/fa saline	fa/fa palm^11^-PrRP31
BW (g)	441 ± 9	572 ± 11****	456 ± 9	592 ± 18***	572 ± 26
Glucose (mmol/l)	4.95 ± 0.13	5.38 ± 0.06**	4.73 ± 0.05	5.29 ± 0.14**	5.91 ± 0.16^#^
TG (mmol/l)	1.11 ± 0.08	4.30 ± 0.28****	1.24 ± 0.15	6.78 ± 1.15***	5.09 ± 0.74
CHOL (mmol/l)	3.37 ± 0.27	7.83 ± 0.51****	2.08 ± 0.15	3.77 ± 0.22****	3.75 ± 0.27
FFA (mmol/l)	0.66 ± 0.04	0.58 ± 0.04	1.79 ± 0.10	1.42 ± 0.18	1.53 ± 0.18
Leptin (ng/ml)	4.02 ± 0.61	46.55 ± 1.61****	3.74 ± 0.51	46.80 ± 1.90****	49.57 ± 2.20
Insulin (ng/ml)	0.49 ± 0.06	1.24 ± 0.02****	0.27 ± 0.06	1.04 ± 0.11***	1.38 ± 0.18
QUICKI			0.374 ± 0.013	0.296 ± 0.005****	0.283 ± 0.004
Liver (g)			15.00 ± 0.65	23.14 ± 0.70****	21.50 ± 0.71
Heart (g)			1.10 ± 0.03	1.19 ± 0.04	1.16 ± 0.03
Kidney (g)			3.18 ± 0.16	3.46 ± 0.16	3.34 ± 0.15
MAP (mmHg)			105.75 ± 6.39	117.67 ± 7.01	125.13 ± 1.57
HR (beat/min)			389.05 ± 13.00	371.07 ± 38.80	378.80 ± 16.37

Data are presented as means ± SEM. Statistical analysis was performed by *t*-test or one-way ANOVA (32 weeks).

Significance is *P < 0.05, **P < 0.01, ***P < 0.001, and ****P < 0.0001 by *t*-test fa/fa vs. control (*n* = 7–8 before the treatment) and one-way ANOVA fa/fa saline vs. control saline (*n* = 7–8 at the end of experiment), ^#^*P* < 0.05 fa/fa palm^11^-PrRP31 vs. fa/fa saline (*n* = 8 at the end of experiment).

**Fig. 2:**
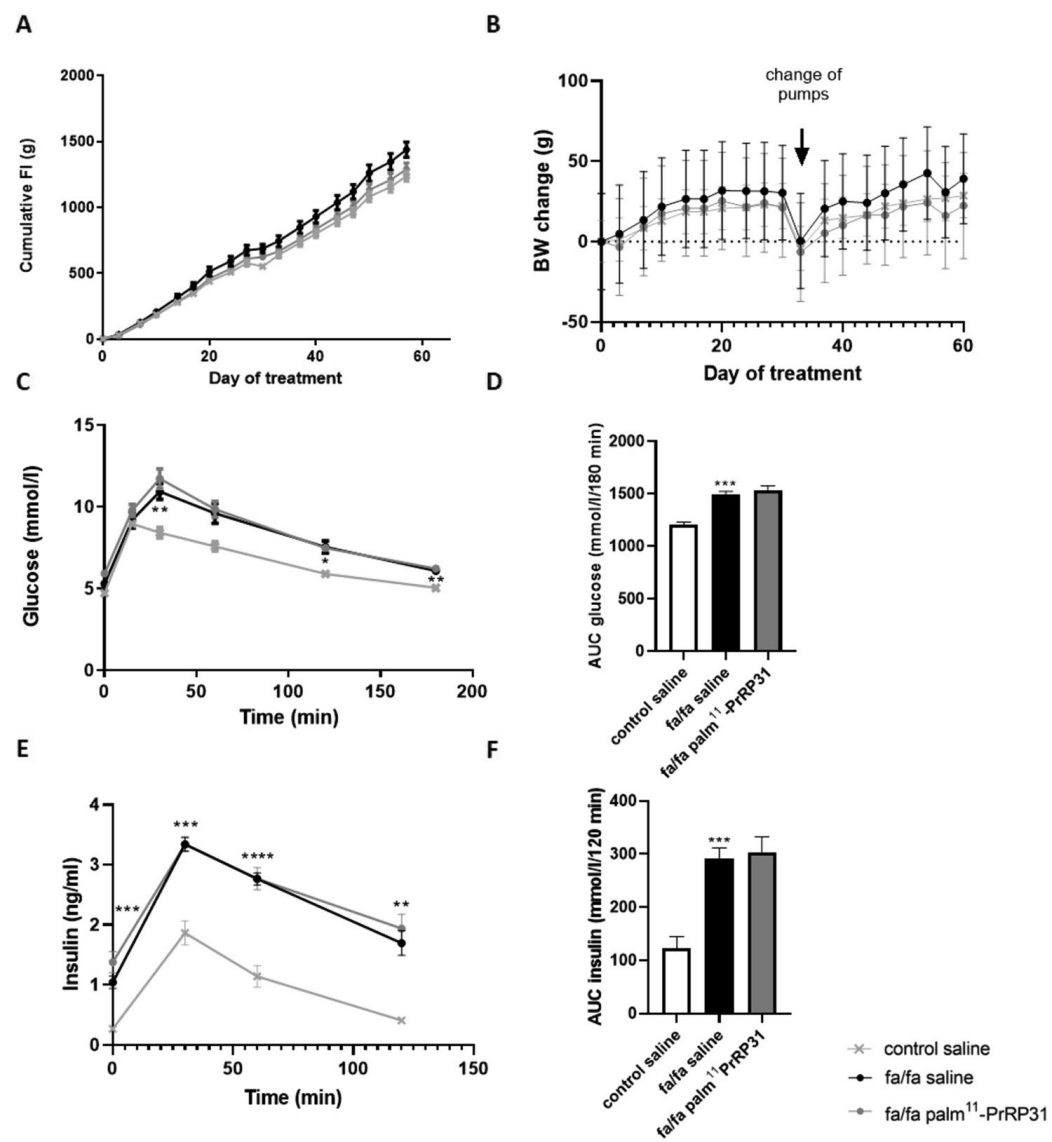
Effect on morphometric and metabolic parameters FI (**A**) and BW **(B**) measured during the treatment of control saline, fa/fa saline, and fa/fa palm^11^-PrRP31 rats. Glucose (**C**, **D**) and insulin (**E**, **F**) measured during the OGTT performed at the end of experiment. Data are presented as means ± SEM. Statistical analysis of AUC was performed by One-way ANOVA. Statistical analysis of BW change, FI, glucose, and insulin was performed by two-way ANOVA. Significance is *P < 0.05, **P < 0.01, ***P < 0.001, and ****P < 0.0001 fa/fa saline vs. control saline (*n* = 7–8).

No significant change in FI was registered among control saline, fa/fa saline, or fa/fa palm^11^-PrRP31 rats during the treatment (Fig. 2A). Similarly, the change in BW over the course of the treatment did not differ among the groups (Fig. 2B). As indicated by the OGTT, the glucose level was significantly higher at 30, 120, and 180 min in the fa/fa saline group than in the control saline group (Fig. 2C), resulting in a significantly increased area under the curve AUC of the fa/fa saline group (Fig. 2D). Palm^11^-PrRP31 treatment did not affect glucose tolerance in fa/fa rats (Fig. 2C, D). The results of the OGTT showed that the fa/fa saline group exhibited a significantly higher plasma insulin level than the control saline group; the insulin levels of the fa/fa palm^11^-PrRP31 group and the fa/fa saline group did not differ (Fig. 2E).

### Palm^11^-PrRP31 attenuated IR and sporadic neuroinflammation and increased mildly synaptogenesis in fa/fa rats

**Fig. 3:**
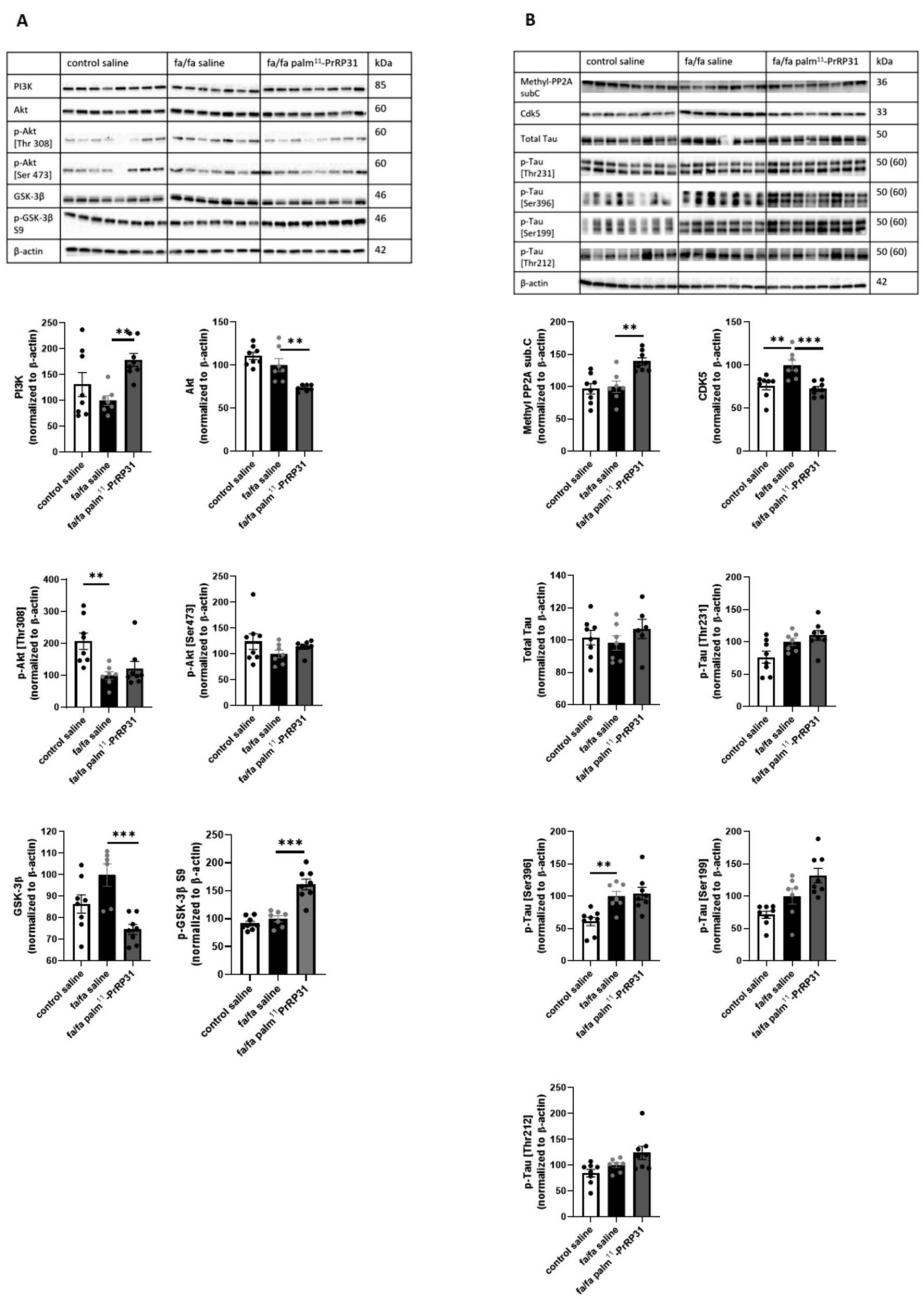
Insulin signaling pathway (**A**), Tau phosphorylation (**B**) measured in hippocampi by western blot at the end of experiment. Data are presented as means ± SEM. Statistical analysis was performed by one-way ANOVA. Significance is *P < 0.05 and **P < 0.01 fa/fa saline vs. control saline (*n* = 7–8).

WB was performed with the hippocampi of control saline, fa/fa saline and fa/fa palm^11^-PrRP31 rats (*n* = 7–8 rats per group). The fa/fa saline group showed a trend toward a decrease in phosphoinositide 3-kinases (PI3K), pAkt (Ser473), and pGSK-3β (Ser9) levels and a significantly lower pAkt (Thr308) level compared to the control saline group suggesting IR in the hippocampus. Palm^11^-PrRP31 treatment significantly increased PI3K and pGSK3β (Ser9) levels, and there was a trend toward an increase in pAkt (Thr308) and pAkt (Ser473) levels (Fig. 3A).

There was non-significant change in methyl-PP2A subC level, but the Cdk5 level was significantly increased in the fa/fa saline group compared to the control saline group. The fa/fa saline group showed non-significant increase in pTau (Thr231), pTau (Ser199) and pTau (Thr212) levels, and a significant increase in pTau (Ser396) levels, compared to the control saline group. Palm^11^-PrRP31 treatment significantly decreased Cdk5 and significantly increased methyl-PP2A subC, but it did not affect Tau phosphorylation at any epitope measured (Fig. 3B).

**Fig. 4:**
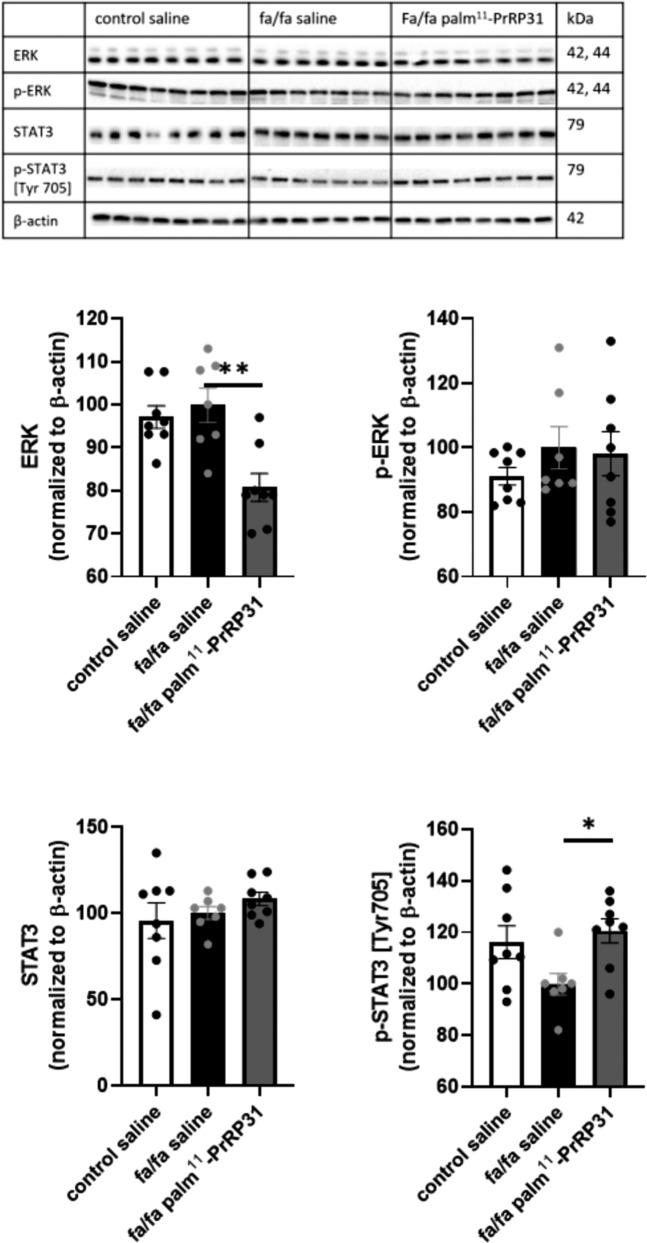
ERK and STAT3 signaling pathway measured in hippocampi by western blot at the end of experiment. Data are presented as means ± SEM. Statistical analysis was performed by One-way ANOVA. Significance is *P < 0.05 (*n* = 7–8).

In the extracellular signal-regulated kinases (ERK) and signal transducer and activator of transcription 3 (STAT3) signaling pathways, no significant differences in phosphorylation were found between the fa/fa saline group and the control saline group but a trend toward a decrease in STAT3 phosphorylation at Tyr705 was evident. STAT3 phosphorylation at Tyr705 was increased in the palm^11^-PrRP31-treated fa/fa rat group compared to the fa/fa saline group (Fig. 4).

**Fig. 5:**
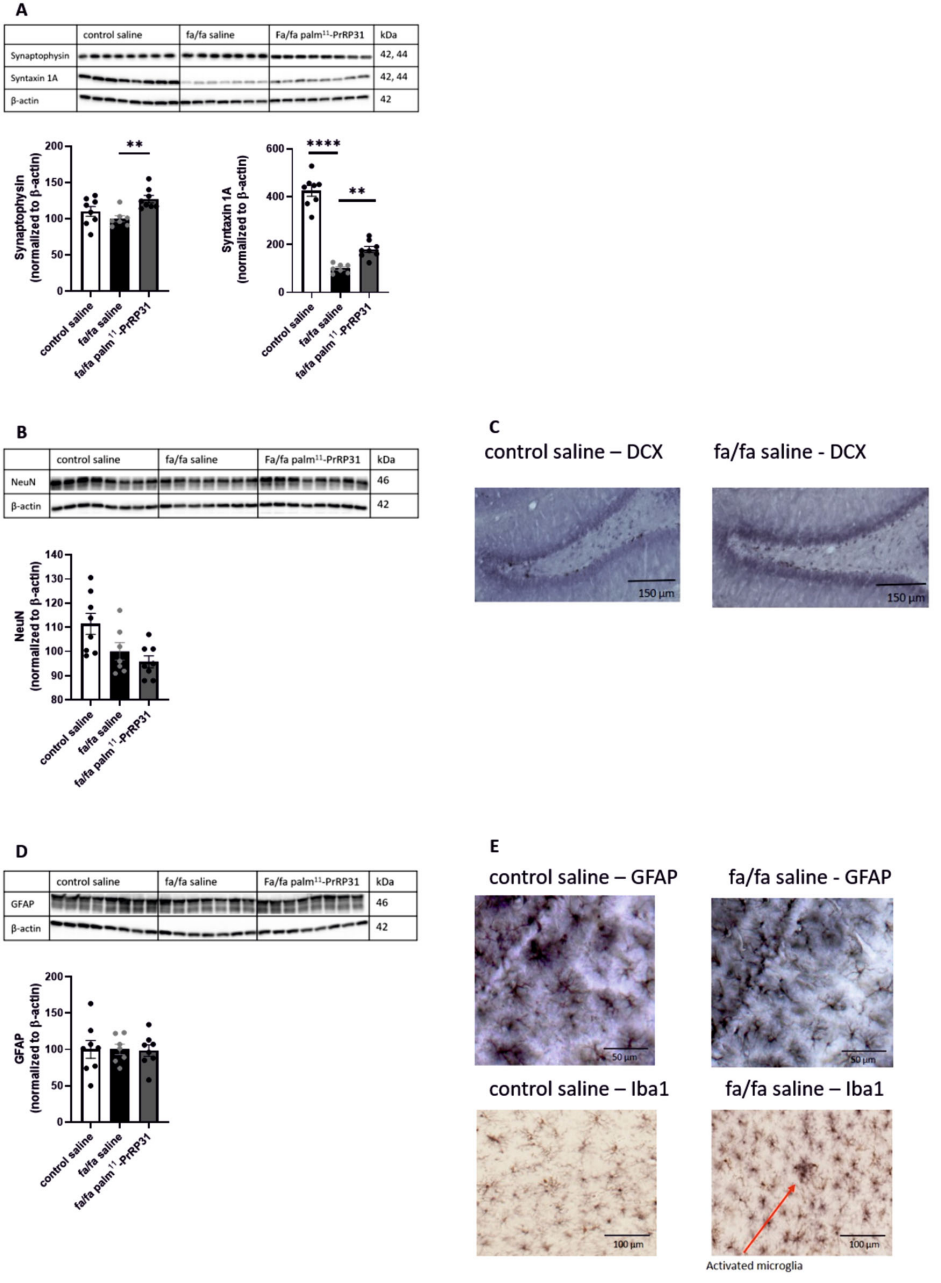
Synaptogenesis and neuroimflammation in brain. Synaptophysin and syntaxin 1A as a marker of synaptogenesis (**A**) and neuronal marker NeuN (**B**) in hippocampi detected by western blot. Marker of neurogenesis doublecortin in hippocampi (**C**) detected immunohistochemically. Marker of neuroinflammation GFAP detected by western blot (**D**) and representative photomicrograph of neuroinflammatory markers GFAP and Iba1 (**E**) at the end of experiment in control saline and fa/fa saline. Data are presented as means ± SEM. Statistical analysis was performed by One-way ANOVA. Significance is *P < 0.05, **P < 0.01, ***P < 0.001, and ****P < 0.0001 (*n* = 7–8).

Synaptogenesis in the hippocampus was detected *via* the presynaptic markers synaptophysin and Syntaxin 1A. Compared to the level in the control saline group, synaptophysin showed a trend toward a decrease in the fa/fa saline group, and Syntaxin 1A was significantly lower in the fa/fa saline group. Both the presynaptic markers synaptophysin and Syntaxin 1A in the hippocampus were significantly increased in the fa/fa palm^11^-PrRP31 group compared to the fa/fa saline group (Fig. 5A).

NeuN is a neuronal marker; there was no significant change between the fa/fa saline and control saline group (Fig. 5B). Moreover, treatment with palm^11^-PrRP31 had no effect on the neuronal marker NeuN. There were no significant changes in the number of DCX^+^ positive cells in the granular layer of the dentate gyrus (DG) (Fig. 5C) as well as in GFAP, a marker of astrocytosis, in the hippocampus of the control saline and fa/fa saline rats as detected by both IHC and by WB (Fig. 5D, E). There was no difference in hippocampal GFAP level in the fa/fa palm^11^-PrRP31 and fa/fa saline rats. IHC staining with the anti-Iba1 antibody showed a slight indication of activated microglia in the fa/fa saline group compared to the control saline group, mainly in the CA1 and DG regions of the hippocampus (Fig. 5E). However, these changes were not significant.

## Discussion

Obese Zucker rats develop the following symptoms of metabolic syndrome: obesity, hyperinsulinemia, IR, glucose intolerance, hyperlipidemia and arterial hypertension [36]. The recessive mutant gene “fatty” (*fa*) of the leptin receptor is the reason for the impaired leptin signaling [8] and extreme obesity with juvenile onset [12].

The design of this study was based on our previous study with 12- and 33-week-old male fa/fa rats [16], where age- and obesity-induced peripheral IR under normoglycemia was described and linked to central IR and pathological Tau hyperphosphorylation.

Therefore, in this study, we presumed that at age of 32 weeks, all these pathologies would be present. We also aimed to determine whether chronic treatment with a palmitoylated PrRP analog administered peripherally can influence obesity and IR as well as Tau phosphorylation, neurogenesis, synaptogenesis, and neuroinflammation in the hippocampus, as it is the first brain structure affected by neurodegenerative changes.

The main difference between the two studies was the source of rats used: in our previous study [16], we used fa/fa rats obtained from Harlan, now Envigo (Italy), and in the present study, we used fa/fa rats obtained from Charles River (Italy).

In obese Zucker fa/fa rats obtained from Envigo, obesity, IR, and hyperlipidemia were reported at 12- [37] or 16 weeks old [38]. In our previous study [16], the Envigo fa/fa rats developed obesity accompanied by significantly increased plasma insulin, glucose intolerance, and IR at 33 weeks of age. In the present study with Charles River rats, these parameters were much less pronounced. Obesity in the Charles River fa/fa rats at 32 and 40 weeks of age was accompanied by mild glucose intolerance and increased insulin compared to the controls; however, insulin level did not reach the same values as in the fa/fa rats from Envigo, and also QUICKI showing peripheral IR was not as decreased, even it was significantly decreased compared to control saline group [16]. Moreover, the Charles River fa/fa rats in this study were not hypertensive. However, elevated blood pressure was reported in Envigo fa/fa rats older than 16 weeks [38], and in 22-week-old fa/fa rats [36] but not in Charles River fa/fa rats [39]. The strain variability in the Charles River and Envigo obese fa/fa rats was previously identified in 8-week-old fa/fa rats and their lean controls. Total lipid, TG, and glucose levels were higher in Charles River rats, while BW, CHOL and insulin levels were higher in the Harlan rats [40]. In our present study, the Charles River fa/fa rats at 32 and 40 weeks of age had a higher plasma TG level but lower BW, CHOL, glucose, and insulin levels than the Envigo rats of a similar age examined in our previous study [16]. We can speculate that fa/fa rats from Charles River used in this study with milder phenotype were too young to develop neurodegenerative features and that older animals could have more pronounced phenotype concerning both metabolic disturbances and neurodegeneration. There is also a possibility that because metabolic disturbances were only weakly developed in fa/fa rats from Charles River, the effect of palm^11^-PrRP31 did not appear clearly in this model.

In our previous study with ZDF rats, we found that intact leptin signaling was necessary for the full effect of palm-PrRP31 on metabolic parameters such as BW-lowering or glucose tolerance; therefore, its antidiabetic and anti-obesity effects were not realized [32]. In Koletsky (spontaneously hypertensive obese, SHROB) rats with a nonsense mutation in the leptin receptor, palm^11^-PrRP31 improved glucose tolerance, but its effect on BW change was minimal [31]. On the other hand, in rat models of high-fat diet-induced obesity that have functioning leptin receptors, such as Sprague-Dawley rats [32] and Wistar Kyoto rats [27], treatment with palmitoylated PrRP significantly decreased BW and blood glucose, as indicated by OGTTs. In this study, treatment with palm^11^-PrRP31 neither affected BW nor attenuated tolerance to glucose. This outcome could have been a result of disrupted leptin receptor signaling in the fa/fa rats, similar to our aforementioned studies with ZDF and Koletsky rats.

Obesity, IR, hyperinsulinemia, and hypertension are features associated with an increased risk of AD, vascular dementia, and impaired cognitive function [20]. In our previous study, we demonstrated that phosphorylation of hippocampal Tau protein at Ser396 and Thr231 was significantly increased in 33-week-old fa/fa rats compared to age-matched controls, and this is a possible consequence of peripheral IR and decreased insulin signaling in the hippocampus [16]. As a consequence of lower obesity and mild peripheral IR observed in the Charles River fa/fa rats compared to the Envigo fa/fa rats manifested by decreased QUICKI, there was only a mild impairment of hippocampal insulin signaling. There was a significant decrease in pAkt (Thr308) and a trend toward a decrease in PI3K and pAkt (Ser473) levels in the fa/fa saline rats compared to the control saline rats. Additionally, we observed a slight decrease in phosphorylation of GSK-3β at inhibitory epitope Ser9, which led to increased kinase activity toward Tau protein. Moreover, Tau kinase Cdk5 was significantly increased; thus, we observed a significant increase in the phosphorylation of Tau at Ser199 and Ser396, both of which had been previously connected to the early stages of neurodegeneration [41, 42]. After the 2-month-long treatment with palm^11^-PrRP31, we observed ameliorated activation of the hippocampal insulin signaling cascade, resulting in significantly increased phosphorylation of GSK-3β at Ser9, which showed inhibited kinase activity toward the Tau protein. We also observed a decrease in the levels of Cdk5, another important Tau kinase. Moreover, activation of methyl-PP2A subC, which is a major Tau phosphatase, was increased. However, Tau hyperphosphorylation at three measured epitopes was not affected; this could have been a result of defective leptin signaling or an insufficient duration of the treatment with palm^11^-PrRP.

It has also been previously shown that intact leptin signaling in the hypothalamus was important for the anorectic effect of leptin in fa/fa rats [43] and proper hippocampal leptin signaling manifested by activation of STAT3, which further can activate Akt, acts as neuroprotective agent and increase survival of hippocampal neurons [44], as well as support memory formation [45]. In concordance with these observation, increased phosphorylation of STAT3 at Tyr705 after the treatment of fa/fa rats with palm^11^-PrRP31 was observed in hippocampus pointing to potential neuroprotective properties of the compound. This finding confirms our previous studies [28, 31], where palmitoylated PrRP analogs activated STAT3 and ERK signaling.

Hippocampal synaptogenesis was reduced in db/db mice lacking functional leptin receptors by an ERK-dependent pathway [46]. In our study, a reduction in synaptogenesis was shown via significantly decreased presynaptic marker Syntaxin 1A in the fa/fa saline rats compared to the saline control rats, however, no changes were observed in hippocampal activation of ERK. Similar to db/db mice previously studied, the levels of synaptophysin and the neuronal marker NeuN did not differ [47]. Moreover, age-related changes linked to metabolic syndrome were described in the cortex and hippocampus of 12-, 16- and 20-week-old fa/fa rats from Envigo [23]; a decrease in a number of neurons, enhancement of gliosis, disruption of the blood brain barrier, and cognitive alterations. In addition, the number of GFAP-immunoreactive astrocytes have been found to be increased in the hippocampi and cortex of fa/fa rats [37]. We observed no changes in astrocytosis and only sporadic signs of micro gliosis in the fa/fa rats. These outcomes could be results of the moderate metabolic syndrome found in the fa/fa rats from Charles River with only mild obesity, and peripheral and central IR [20]. Stranahan et al. [15] described decreased neurogenesis in db/db mice using BrdU staining; in our study, we did not observe any changes in neurogenesis in the hippocampus of the fa/fa saline rats compared to the control rats using DCX.

From all the results obtained from this and previous studies, it is possible to summarize that palm^11^-PrRP31 can ameliorate neurodegeneration by an increase of insulin and leptin signaling, as well as by increase of synaptogenesis and neurogenesis in brain of treated animals.

In conclusion, this study demonstrates that fa/fa rats from Charles River developed obesity, mild glucose intolerance, and mild central and peripheral IR but not hypertension. Mildly worsened synaptogenesis with no difference in neurogenesis and sporadic neuroinflammation was observed in the fa/fa saline rats compared to the saline controls. Eight-week treatment with palm^11^-PrRP31 neither lowered BW nor attenuated glucose tolerance, suggesting that intact leptin signaling was necessary for the anti-obesity effect of palm^11^-PrRP31, similar to our previous studies. Treatment with palm^11^-PrRP31 had no effect on Tau phosphorylation or neuroinflammation; however, it ameliorated insulin signaling, increased activation of PP2A, decreased activation of Tau kinases, and increased synaptogenesis in the hippocampus. This mild beneficial effect of palm^11^-PrRP31 treatment was probably due to the lack of leptin receptor activity, but despite of no improvement in metabolic profile, some neuroprotective effects were observed. These results indicate the preserved neuroprotective effects of palm^11^-PrRP despite of disrupted leptin signaling in this rat model.

## Supplementary information

Supplementary Table 1
