# Supplementary material for: Palmitoylated prolactin-releasing peptide treatment had neuroprotective but not anti-obesity effect in fa/fa rats with leptin signaling disturbances
Source: Nutr Diabetes. 2022 May 19;12:26. doi: 10.1038/s41387-022-00205-3 (PMC9119973; doi:10.1038/s41387-022-00205-3)
Supplement: Supplementary file 1 — Supplementary Table 1 [file 41387_2022_205_MOESM1_ESM.docx]

**Supplementary Table 1**

| **Antibody** | **Manufacturer** | **WB dilution** |
| --- | --- | --- |
| **PI3K** | Cell Signaling Technology, Beverly, MA, USA | 1:1000 5% BSA TBS/tween-25 |
| **total Akt** | Cell Signaling Technology, Beverly, MA, USA | 1:1000 5% milk TBS/tween-22 |
| **pAkt (Thr308)** | Cell Signaling Technology, Beverly, MA, USA | 1:1000 5% milk TBS/tween-23 |
| **pAkt (Ser473)** | Cell Signaling Technology, Beverly, MA, USA | 1:1000 5% milk TBS/tween-24 |
| **GSK-3β** | Cell Signaling Technology, Beverly, MA, USA | 1:1000 5% BSA TBS/tween-20 |
| **pGSK-3β (Ser9)** | Cell Signaling Technology, Beverly, MA, USA | 1:1000 5% BSA TBS/tween-21 |
| **Methyl-PP2A subC** | Sigma, St. Louis, MO, USA | 1:1000 5% BSA TBS/tween-40 |
| **Cdk5** | Sigma, St. Louis, MO, USA | 1:2000 5% BSA TBS/tween-39 |
| **Total Tau N ter** | (Tau N ter, gift from Dr. M.-C. Galas, INSERM U837, Lille, France) | 1:5000 5% milk TBS/tween-32 |
| **pTau (Thr231)** | Invitrogen Grand Island, NY, USA | 1:10000 5% BSA TBS/tween-33 |
| **pTau (Ser396)** | Invitrogen Grand Island, NY, USA | 1:10000 5% BSA TBS/tween-34 |
| **pTau (Ser199)** | Thermo Fisher Rockford, IL, USA | 1:1000 5% BSA TBS/tween-35 |
| **pTau (Thr212)** | Thermo Fisher Rockford, IL, USA | 1:1000 5% BSA TBS/tween-36 |
| **ERK** | Cell Signaling Technology, Beverly, MA, USA | 1:1000 5% milk TBS/tween-26 |
| **pERK** | Cell Signaling Technology, Beverly, MA, USA | 1:1000 5% milk TBS/tween-27 |
| **STAT3** | Cell Signaling Technology, Beverly, MA, USA | 1:1000 5% milk TBS/tween-28 |
| **pSTAT (Tyr705)** | Cell Signaling Technology, Beverly, MA, USA | 1:1000 5% milk TBS/tween-29 |
| **Synaptophysin** | Santa Cruiz | 1:5000 5% milk TBS/tween-38 |
| **Syntaxin1A** | Cell Signaling Technology, Beverly, MA, USA | 1:1000 5% BSA TBS/tween-30 |
| **NeuN** | Thermo Fisher Rockford, IL, USA | 1:1000 5% BSA TBS/tween-37 |
| **GFAP** | Cell Signaling Technology, Beverly, MA, USA | 1:1000 5% milk TBS/tween-31 |
| **β-actin** | Sigma, St. Louis, MO, USA | 1:10000 5% milk TBS/tween-41 |

**Supplementary Table 1 List of primary antibodies and their appropriate dilution used for western blot.**

PI3K total phosphoinositide 3-kinase, ERK extracellular signal-regulated kinase 1/2, STAT3 total signal transducer and activator of transcription 3, GFAP glial fibrillary acidic protein, Cdk5 cyclin-dependent kinase 5, methyl-PP2A subC methyl protein phosphatase 2A subunit C
